# 

**Published:** 1908-05

**Authors:** 


### CONTENTS.

ORIGINAL COMMUNICATIONS—
Weaning. By H. McHatton, M. D., Macon, Ga. .. 59
Early Operation for Adenoids. By Alex W. Stirling, M. D., C. M., (Edin.) D. P. H. (London). Oculist and aurist to the Wesley Memorial, Presbyterian and Tabernacle Hospitals, and to the Atlanta, Birmingham and Atlanta Railway Company, Atlanta, Ga. .. 68
Hydrocele and Spermatocele, with Report of Cases. By W. L. Champion, M. D., Atlanta, Ga. .. 71
Treatment of Sprains. By Theodore Toepel, M. D., Lecturer on Medical Gymnastics at the Atlanta College of Physicians and Surgeons, Director of Physical education in the Atlanta Public Schools, Medical Examiner at the Atlanta Athletic Club .. 74
Crushes of the Extremities. By Thos. H. Hancock, M. D., Atlanta, Ga. .. 77
The Importance and Scope of Remedies Increasing the Activity of the Lymphatics, with Special Reference to Iodalbin. By Noble M. Elerhart, M. S., M. D. .. 80
EDITORIALS—
Early Diagnosis of Pulmonary Tuburculosis .. 86
Magic Healer, not a Physician! ! .. 88
To Prevent Ophthalmia Neonatiorum .. 90
Liberal and Sensible Construction of the Prohibition Law .. 91
The Mosquito Pest .. 91
The Papers at the Recent Meeting of the Medical Association of Georgia .. 92
NEWS AND NOTES—
The Medical Association of Georgia .. 94
Mississippi Valley Medical Association .. 94
Names and Addresses of the Secretaries of the Various State Examining Boards .. 108
Fifth Pan-American Medical Congress .. 110
Notice to Alumni of the Tulane Medical Department .. 111
Regular Meeting, Fulton County Medical Society, April 2, 1908. Edited by R. R. Daly, M. D. .. 112
Fulton County Medical Society, March 19 .. 113
BOOK REVIEWS .. 114
SELECTIONS AND ABSTRACTS .. 120

